# Novel mutations in ADAMTS13 CUB domains cause abnormal pre‐mRNA splicing and defective secretion of ADAMTS13

**DOI:** 10.1111/jcmm.15025

**Published:** 2020-02-19

**Authors:** Yizhi Jiang, Dongping Huang, Yuji Kondo, Miao Jiang, Zhenni Ma, Lu Zhou, Jian Su, Xia Bai, Changgeng Ruan, Zhaoyue Wang, Lijun Xia

**Affiliations:** ^1^ Department of Hematology The Affiliated Yijishan Hospital of Wannan Medical College Wuhu China; ^2^ Key Laboratory of Thrombosis and Hemostasis of Ministry of Health Jiangsu Institute of Hematology The First Affiliated Hospital of Soochow University Suzhou China; ^3^ Collaborative Innovation Center of Hematology Soochow University Suzhou China; ^4^ Cardiovascular Biology Research Program Oklahoma Medical Research Foundation Oklahoma City OK USA; ^5^ Department of Hematology Affiliated Hospital of Nantong University Nantong China; ^6^ State Key Laboratory of Radiation Medicine and Protection Soochow University Suzhou China

**Keywords:** ADAMTS13, genetic mutations, thrombotic thrombocytopenic purpura, von Willebrand factor

## Abstract

Hereditary thrombotic thrombocytopenic purpura (TTP) is an autosomal recessive thrombosis disorder, caused by loss‐of‐function mutations in *ADAMTS13*. Mutations in the CUB domains of *ADAMTS13* are rare, and the exact mechanisms through which these mutations result in the development of TTP have not yet been fully elucidated. In this study, we identified two novel mutations in the CUB domains in a TTP family with an acceptor splice‐site mutation (c.3569−1, G>A, intron 25) and a point missense mutation (c.3923, G>A, exon 28)*,* resulting in a glycine to aspartic acid substitution (p.G1308D). In vitro splicing analysis revealed that the intronic mutation resulted in abnormal pre‐mRNA splicing, and an in vitro expression assay revealed that the missense mutation significantly impaired ADAMTS13 secretion. Although both the patient and her brother displayed significantly reduced ADAMTS13 activity and increased levels of ultra‐large VWF (ULVWF) multimers in plasma, only the female developed acute episodes of TTP. Our findings indicate the importance of the CUB domains for the protein stability and extracellular secretion of ADAMTS13.

## 1 INTRODUCTION

Hereditary thrombotic thrombocytopenic purpura (TTP) is caused by a severe deficiency in the activity of the plasma ADAMTS13 (A disintegrin‐like and metalloproteinase with a thrombospondin type 1 motif, member 13), resulting from loss‐of‐function mutations in *ADAMTS13.* 1 TTP is a rare yet life‐threatening disorder, primarily characterized by severe thrombocytopenia, microvascular haemolytic anaemia and multiorgan damage.^2^ ADAMTS13 specifically regulates the size of von Willebrand factor (VWF) multimers by cleaving the peptide bond between tyrosine and methionine in the exposed VWF A2 domain, preventing the development of microvascular thrombosis.^3^

**Figure 1:**
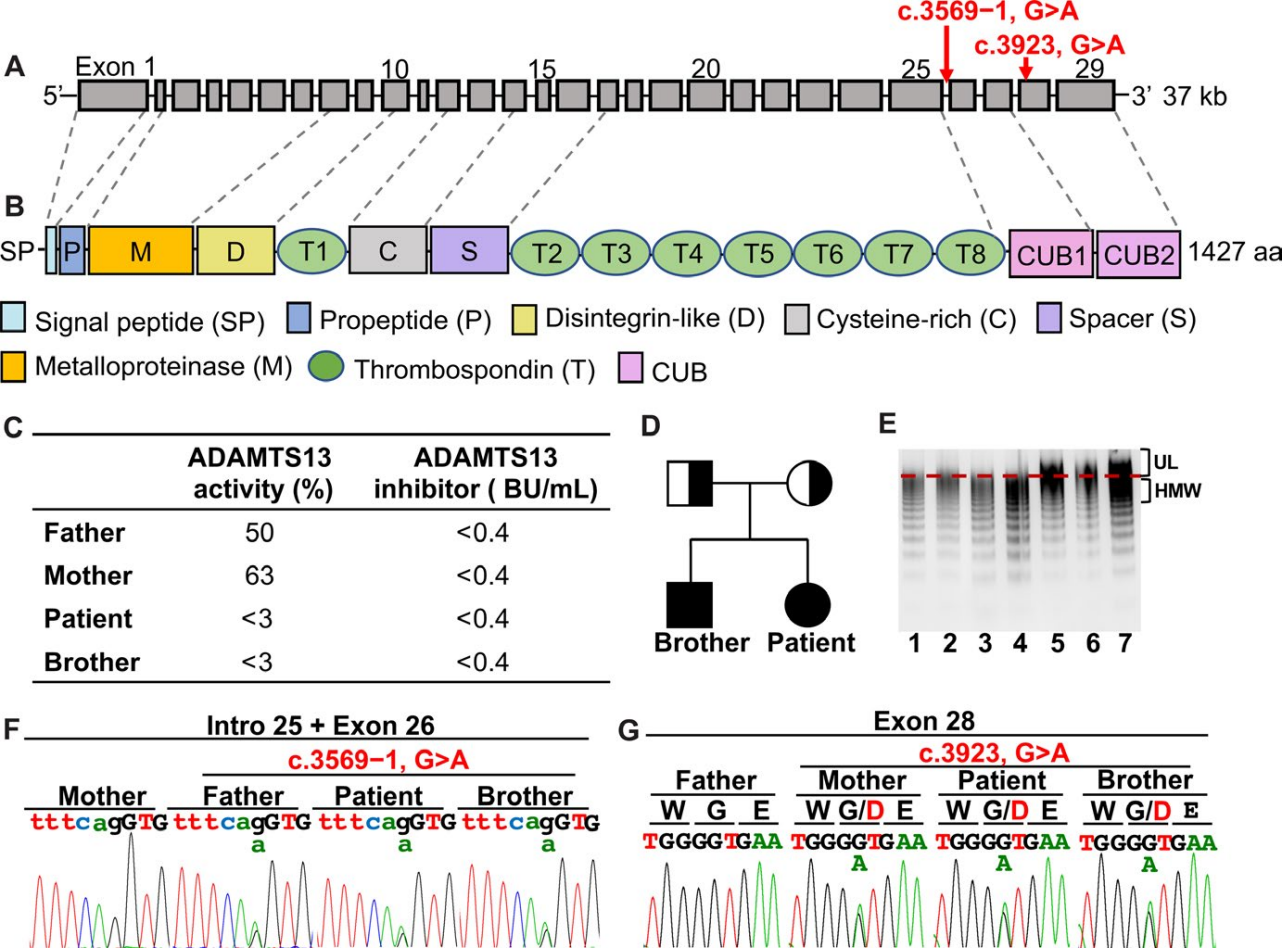
Structures of the ADAMTS13 gene and protein, and ADAMTS13‐related laboratory and genetic profiles of the patient and her family members. A, The *ADAMTS13,* localized on chromosome 9q34, contains 29 exons. The dashed lines indicate the correspondence between *ADAMTS13* exons and ADAMTS13 protein domains. B, The functional domains in the ADAMTS13 protein, from the N‐terminus to the C‐terminus, have 1427 amino acids. C, ADAMTS13 activity and inhibitor of the family. D, Family pedigree. Filled circle or square, compound heterozygotes; half‐filled square or circle, heterozygote. E, VWF multimer patterns observed in plasma. Lanes 1 and 2, normal plasma; Lane 3, father; Lane 4, mother; Lanes 5 and 6, patient (different plasma volumes); and Lane 7, brother. Ultra‐large (UL) VWF multimers are at the top, high‐molecular‐weight (HMW) multimers are at the bottom. Plasma samples of the patient and her brother show ULVWF multimers. F, An acceptor splice‐site mutation, located in intron 25 (3569−1G>A, intron 25), was detected in the patient, her father and her brother. G, A point missense mutation in the CUB2 domain (3923G>A, exon 28), leading to a p.G1308D substitution, was detected in the patient, her mother and her brother

Hereditary TTP is inherited through an autosomal recessive pattern, comprises <5% of all TTP cases^4^ and is associated with bi‐allelic mutations in *ADAMTS13* that result in the absence or severe deficiency of ADAMTS13 protein and activity.^1^ ADAMTS13 is an approximately 190‐kD multidomain protein, consisting of proximal metalloprotease, disintegrin‐like, cysteine‐rich and spacer domains, as well as thrombospondin domains, and two CUB (complement components C1r/C1s, Uegf and bone morphogenic protein 1) domains (Figure 1A,B).^5^ The CUB domains are unique to ADAMTS13, among the members of the ADAMTS family, and are composed of 10 β‐strands, arranged into two β‐sheets that are stabilized by two disulphide bonds.^6^

To date, more than 100 mutations in *ADAMTS13* have been reported.^7^ However, mutations in the CUB domains are rare, and how these mutations affect the biosynthesis and function of ADAMSTS13 remains unclear.^8^ We discovered two novel mutations in the ADAMSTS13 CUB domains in a sibling pair who displayed significantly reduced ADAMTS13 activity, although only the female developed acute TTP episodes. In vitro studies revealed that the mutations affected the mRNA splicing and protein secretion of ADAMTS13.

## 2 MATERIALS AND METHODS

### 2.1 Patient

The proband was a 31‐year‐old woman. Her blood and her family member's blood were collected to tubes coated with ethylenediaminetetraacetic acid (EDTA) or containing 3.2% of sodium citrate. This study was approved by the Ethic Committee of the First Affiliated Hospital of Soochow University. All participants provided informed consent.

### 2.2 Genomic DNA analysis

Genomic DNA was isolated from peripheral blood leucocytes using a GenElute Blood Genomic DNA Kit (Millipore Sigma). All 29 exons and intron‐exon boundaries of *ADAMTS13* were sequenced by Next‐generation sequencing (NGS) on NextSeq500 platform (Illumina). The regions containing abnormal sites identified by NGS were amplified by polymerase chain reaction (PCR) and then sequenced on an ABI 3130XL Genetic Analyzer (Applied Biosystems). Sequencing results were compared against the reference sequence for *ADAMTS13* (NCBI: NC_000009.11).

### 2.3 Assay of plasma ADAMTS13 activity and inhibitor

To obtain plasma, whole‐blood samples were centrifuged at 1000 *g*, for 15 minutes at 4°C. A modified FRETS‐VWF73 (Peptides International) assay^9^ and a chromogenic ADAMTS13 activity enzyme–linked immunosorbent assay (Kainos Laboratories Inc)^10^ were performed to measure the levels of ADAMTS13 activity and inhibitor in the plasma.

### 2.4 VWF multimer analysis

Plasma samples were separated by 1.3% SDS Seakem Gold agarose (LONZA) gel electrophoresis and Western blotted under non‐reducing conditions.^11^ The gel was first incubated with rabbit anti‐human vWF antibody (DAKO) and horseradish peroxidase (HRP)‐labelled goat anti‐rabbit IgG (Thermo Fisher Scientific). The gel was then incubated with a chemiluminescence substrate (SuperSignal West Pico chemiluminescent substrate kit, Thermo Fisher Scientific) and exposed to X‐ray films (Kodak).

### 2.5 In vitro mini‐gene expression

Exon 26 (147‐bp) and the flanked introns (150‐bp) of *ADAMTS13* were amplified by PCR using genomic DNA from the patient and a healthy control. The fragments were then cloned into an Exontrap Cloning Vector pET01 (MoBiTec GmbH) for the mini‐gene assay. The *ADAMTS13* vectors or empty control vector was then transfected into COS‐7 cells. After 48 hours of incubation, total RNA was extracted using TRIzol (Millipore Sigma) and genomic DNA was digested by treating with DNase I (Millipore Sigma). Reverse transcription PCR (RT‐PCR) was performed with M‐MLV Reverse Transcriptase (Invitrogen). The PCR products were analysed on a 1.5% agarose gel and sequenced.

### 2.6 Expression of recombinant wild‐type (WT) and p.G1308D mutant ADAMTS13

The WT ADAMTS13 cDNA was cloned into the mammalian expression vector pSecTag2/Hygro (Invitrogen), and the p.G1308D mutant was then generated, by site‐directed mutagenesis. The WT and mutant constructs were then transiently expressed in COS‐7 cells. The serum‐free conditioned medium was collected at 48 hours after transfection, and secreted polyhistidine‐tagged ADAMTS13 was purified by Ni sepharose (GE Healthcare). Cells were lysed in a RIPA buffer (50 mmol/L Tris‐HCl pH 7.4, 150 mmol/L NaCl, 1% Triton X‐100, 1% Sodium deoxycholate, 0.1% SDS, 1 mmol/L EDTA) supplemented with fresh protease inhibitor (1:100, Thermo Fisher Scientific). Samples were analysed by Western blotting using mouse anti‐polyhistidine antibody (Abcam) or rabbit anti‐GAPDH antibody (Santa Cruz Biotechnology Inc) followed by HRP‐conjugated goat antimouse IgG or goat anti‐rabbit IgG, respectively.

### 2.7 Immunofluorescence microscopy

Cultured COS‐7 cells were transfected with expression constructs, as described in this paper. After 48 hours, the cells were fixed with 2% paraformaldehyde/phosphate‐buffered saline (PBS), permeabilized with 0.2% Triton X‐100/PBS and blocked with 2% bovine serum albumin (BSA)/PBS. Cells were then incubated with mouse anti‐polyhistidine (Abcam) and rabbit anti‐Calnexin (Gene Tex) primary antibodies, followed by fluorescence‐conjugated secondary antibodies (Jackson ImmunoResearch). Counterstaining was performed with 4′,6‐diamidino‐2‐phenylindole (DAPI, Vector Laboratories). Samples were analysed with an Eclipse 80i microscope (Nikon).

## 3 RESULTS

### 3.1 A 31‐year‐old female was diagnosed as hereditary TTP with two novel mutations in CUB domains of ADAMTS13

The proband suffered from dizziness, vomiting and slurred speech during the 14th week of her second gestation, with a history of induced abortion during the 16th week of a previous gestation. Her 29‐year‐old brother and parents did not have any similar symptoms. Upon admission, the level of plasma ADAMTS13 activity of the patient was <3%, without a detectable functional inhibitor in the plasma (Figure 1C). She was immediately treated with fresh‐frozen plasma infusions. After treatment, peripheral blood counts and vital parameters were recovered. NGS analysis revealed two novel compound heterozygous mutations in the CUB domains of *ADAMTS13* with an acceptor splice‐site mutation (c.3569−1, G>A, intron 25) and a missense mutation (c.3923, G>A, exon 28) causing a glycine to aspartic acid substitution in the patient (Figure 1F,G). She was diagnosed as hereditary TTP, and an induced abortion was successfully performed at that time. She was subsequently maintained on regular plasma infusions for 2 months.

### 3.2 The sibling pair has compound heterozygous mutations of ADAMTS13 with reduced ADAMTS13 activity

We isolated the genomic DNA from the family members and sequenced all 29 exons and intron‐exon boundaries in *ADAMTS13*. Further Sanger sequencing analysis confirmed that the patient's brother had the same mutations as her (Figure 1F,G). Their parents each carry one heterozygous mutation (Figure 1D, 1 and G). ADAMTS13 activity assays were performed with fresh plasma. The siblings showed almost undetectable ADAMTS13 activity levels (normal range > 45%), without the presence of any ADAMTS13 functional inhibitors (normal range < 0.4 BU/mL) (Figure 1C). Compared with normal controls and the parents of the sibling pair, VWF multimer analysis of the sibling pair showed increased levels of ULVWF multimers, indicating reductions in VWF proteolysis by ADAMTS13 (Figure 1E).

### 3.3 The c.3569−1 G>A mutation results in an abnormal pre‐mRNA splicing with a premature termination codon (PTC)

**Figure 2:**
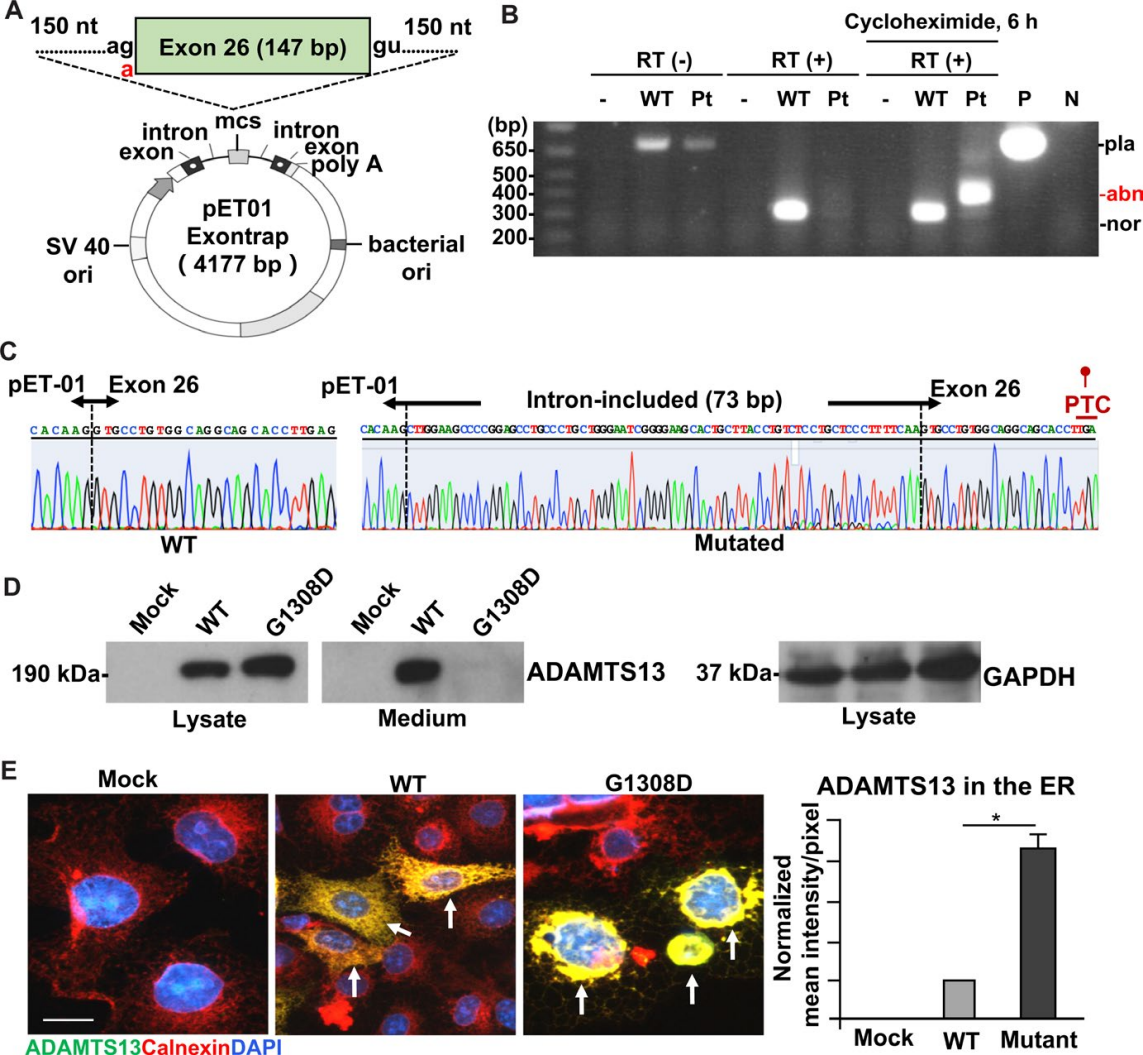
The c.3569−1G>A mutation creates a PTC in exon 26 and the c.3923G>A causes defective secretion of ADAMTS13. A, Designed mini‐gene structure. The mini‐gene contains exon 26 and the flanked 150‐bp introns of *ADAMTS13* genomic DNA. B, WT and patient mini‐gene RT‐PCR products. A 326‐bp band, indicating normal splicing, was produced by WT mini‐gene, whereas the patient mini‐gene showed the same 326‐bp. Cells transfected with the patient mini‐gene also had an additional abnormally spliced band (~400 bp) after treated with cycloheximide as an inhibitor of NMD. RT (−), without reverse transcription; RT (+), with reverse transcription; ‐, empty plasmid; Pt, patient; P, positive control (WT mini‐gene plasmid); N, negative control (water); pla, unspliced plasmid form; abn, abnormally spliced form; nor, normally spliced form. C, Sequence analysis identified a 73‐bp intron inclusion containing a PTC in exon 26 in the patient mini‐gene transcripts. D, Western blot analysis of WT and G1308D mutant of ADAMTS13 protein expressed in COS‐7 cells. GAPDH, loading control. Mock, cells transfected with empty vectors. E, Representative immunofluorescent images of COS‐7 cells transfected with an empty vector or plasmids expressing WT or G1308D mutant ADAMTS13 (Arrows). Bar graphs represent quantification of the ADAMTS13 in the ER. Scale bars, 20 μm. *P < .05

To detect the effects of the G to A mutation at the splice acceptor site of intron 25, we performed an in vitro splicing assay by constructing a mini‐gene expression system because patient mRNA was not available (Figure 2A). RT‐PCR products from the cells transfected with WT mini‐gene showed an approximately 326‐bp band, indicating normal splicing (Figure 2B). Cells transfected with the patient mini‐gene showed the same 326‐bp band as the WT; however, an additional abnormally spliced band (~400‐bp) appeared in the cells after treated with cycloheximide as an inhibitor of non‐sense–mediated mRNA decay (NMD). This result indicates that the abnormally spliced transcript from patient mini‐gene is degraded through NMD (Figure 2B). Therefore, we concluded that the splicing pattern of the patient *ADAMTS13* mRNA is different from that of WT. Further sequence analysis identified a 73‐bp intron inclusion that contains a PTC in exon 26 (Figure 2C).

### 3.4 The G1308D mutation causes an increased accumulation of ADAMTS13 in the ER

To determine the effects of the p.G1308D mutation on ADAMTS13 secretion, COS‐7 cells were transiently transfected with ADAMTS13 expression vectors of WT and p.G1308D constructs. Lysates from G1308D‐transfected cells contained similar amounts of ADAMTS13 as lysates from WT‐transfected cells (Figure 2D). However, in the conditioned media, the amount of p.G1308D protein was almost undetectable, indicating that the mutation affected the secretion or stability of the ADAMTS13 protein. To further investigate the molecular mechanism underlying the ADAMTS13 deficiency caused by the p.G1308D mutation, we next performed immunofluorescent staining to observe the intracellular localization of the WT and mutant ADAMTS13 proteins, together with an ER protein marker, calnexin. In cells expressing recombinant WT ADAMTS13, the protein was evenly distributed in the cytoplasm, in a diffuse pattern (Figure 2E), with limited overlap between ADAMTS13 and calnexin staining. In contrast, in cells expressing the mutant ADAMTS13, the protein primarily accumulated around the nucleus and colocalized with calnexin. These observations indicated that a large amount of the mutant ADAMTS13 molecules were retained in the ER, indicating an impaired extracellular secretion ability.

## 4 DISCUSSION

Our analysis shows the two novel mutations located in the CUB domains of *ADAMTS13* are pathogenic. The c.3569−1 mutation (G>A, intron 25) results in an abnormal pre‐mRNA splicing with PTC, whereas the G1308D mutation causes an increased accumulation of ADAMTS13 in the ER.

Hereditary TTP is an autosomal recessive disorder; men and women would be equally affected by this disease.^4^, ^7^ However, in our study, only the patient had an acute episode of TTP after pregnancy. Studies have shown that in women who had TTP during their first pregnancy, the frequency of hereditary TTP was 25%‐66%.^12^ How pregnancy triggers TTP is unclear. Women with pregnancy are known to have elevated ULVWF multimers compared with the non‐pregnant state.^12^ In this family, although both siblings had ULVWF multimers in plasma and almost undetectable levels of ADAMTS13 activity, the brother has never had acute TTP. More interestingly, the female did not experience any TTP episodes during her first pregnancy, even though her first pregnancy failed during the second trimester. Our results suggested that unknown genetic or environmental factors, such as infections, cytokines, might also contribute to the development of acute TTP.

Mutations at the conserved GT‐AG motif can cause errors during the splicing process, resulting in alterations to the open reading frame (ORF). To confirm the pathogenicity of the intronic variant identified in the CUB domain of the patient, we performed an in vitro splicing assay by a mini‐gene analysis. We found that the intronic variant inactivated the existing splice acceptor site, resulting in a frameshift in the ORF and the introduction of a PTC, which leads to faster mRNA degradation through a protective process NMD. The results of the in vitro splicing assay suggested that the c.3569−1, G>A variant in the CUB domain was pathogenic for severely deficient ADAMTS13 activity.

Unlike the clear roles played by the proximal MDTCS domains of ADAMTS13 during microvascular thrombosis, the specific functions of the distal domains have not been fully clarified. The CUB domains may play crucial roles in the recognition and cleavage of VWF multimers.^13^ Recent studies have demonstrated that the distal T‐CUB domains have allosteric properties, which may markedly inhibit substrate cleavage and be essential for relieving autoinhibition upon binding to VWF.^14^, ^15^ Several studies have shown that mutations in the CUB domains are likely to impair the secretion and/or extracellular degradation of ADAMTS13.^8^, ^16^ We found that the p.G1308D mutation causes an increased accumulation of ADAMTS13 in the ER, which leads to impaired secretion of ADAMTS13. Although how the CUB domain regulates the trafficking of ADAMTS13 in the ER remains to be studied, our results provide new molecular insights into the molecular pathogenesis of the hereditary TTP.

## CONFLICT OF INTEREST

The authors have no conflict of interest.

## AUTHORS' CONTRIBUTIONS

YJ, ZW, CR and LX recruited the patient and designed the project; YJ, DH, MJ, ZM, LZ and XB collected and analysed the clinical data; YJ, YK and JS performed the experiments and analysed data; YJ, DH and LX organized data and wrote the manuscript.

## Data Availability

The data that support the findings of this study are available on request from the corresponding author. The data are not publicly available due to privacy or ethical restrictions.
